# High incidence of cervical ribs indicates vulnerable condition in Late Pleistocene woolly rhinoceroses

**DOI:** 10.7717/peerj.3684

**Published:** 2017-08-29

**Authors:** Alexandra A.E. van der Geer, Frietson Galis

**Affiliations:** 1 Naturalis Biodiversity Center, Leiden, the Netherlands; 2 Department of Geology and Geoenvironment, National and Kapodistrian University of Athens, Zografou, Greece

**Keywords:** Transitional vertebrae, *Coelodonta*, North sea, Developmental constraints, Neck vertebrae, Extinction, Neck ribs, Rhinoceratidae

## Abstract

Mammals as a rule have seven cervical vertebrae, a number that remains remarkably constant. Changes of this number are associated with major congenital abnormalities (pleiotropic effects) that are, at least in humans, strongly selected against. Recently, it was found that Late Pleistocene mammoths (*Mammuthus primigenius*) from the North Sea have an unusually high incidence of abnormal cervical vertebral numbers, approximately ten times higher than that of extant elephants. Abnormal numbers were due to the presence of large cervical ribs on the seventh vertebra, indicating a homeotic change from a cervical rib-less vertebra into a thoracic rib-bearing vertebra. The high incidence of cervical ribs indicates a vulnerable condition and is thought to be due to inbreeding and adverse conditions that may have impacted early pregnancies in declining populations. In this study we investigated the incidence of cervical ribs in another extinct Late Pleistocene megaherbivore from the North Sea and the Netherlands, the woolly rhinoceros (*Coelodonta antiquitatis*). We show that the incidence of abnormal cervical vertebral numbers in the woolly rhinoceros is unusually high for mammals (15,6%, *n* = 32) and much higher than in extant Rhinoceratidae (0%, *n* = 56). This indicates that woolly rhinoceros lived under vulnerable conditions, just like woolly mammoths. The vulnerable condition may well have contributed to their eventual extinction.

## Introduction

The number of cervical vertebrae in mammals is remarkably constant at seven, in contrast to other tetrapods, where this number varies considerably (Leboucq, 1898; Schultz, 1961; Starck, 1979; Narita & Kuratani, 2005). The only exceptions are manatees (*Trichechus*, Sirenia) and extant sloths (*Bradypus* and *Choloepus*, Xenarthra). These latter taxa have an exceptional number of cervical vertebrae (Bateson, 1894; Starck, 1979; Varela-Lasheras et al., 2011). The extreme evolutionary conservation of the number of cervical vertebrae in mammals implies that there must be selection against intraspecific variation of this number. Yet intraspecific variation does occur. The most common variation is a partial or complete homeotic transformation of a cervical into a thoracic vertebra (involving a change in the activity of *Hox* genes) in the form of the presence of ribs on the seventh vertebra, so-called cervical ribs (Galis, 1999; Li & Shiota, 2000; Varela-Lasheras et al., 2011; Wéry et al., 2003). These cervical ribs are usually rudimentary and not fused to the sternum, but they may be fused to the first rib instead. In humans, remarkably strong selection against such cervical ribs was shown to exist (Galis, 1999; Galis et al., 2006; Furtado et al., 2011; Ten Broek et al., 2012). Approximately 90% of individuals with a cervical rib die before reaching reproductive age (Galis et al., 2006). The cervical rib itself is relatively harmless, but its presence is associated with multiple and major congenital abnormalities (Galis et al., 2006; Ten Broek et al., 2012). This is because its development is induced by a (genetic or environmental) disturbance of early embryogenesis (Li & Shiota, 2000; Wéry et al., 2003; Chernoff & Rogers, 2004; Galis et al., 2006). Usually, such a disturbance has multiple effects, due to the highly interactive nature of early embryogenesis. Hence, the strong selection against cervical ribs is indirect and due to the severity of the associated medical problems (Galis et al., 2006; Ten Broek et al., 2012). Indeed, we have found an association with abnormalities in other mammalian species as well (Varela-Lasheras et al., 2011). Even in the exceptional manatees and sloths, support was found for an association between abnormal numbers of cervical vertebrae and severe skeletal abnormalities, including fused vertebrae and ossification defects. The selection against these abnormalities is apparently sufficiently relaxed in these latter taxa to allow for the breaking of the constraint on changes of the number of cervical vertebrae (Varela-Lasheras et al., 2011).

An unusually high incidence of cervical ribs (33.3%) was found in the woolly mammoth *Mammuthus primigenius* (Blumenbach, 1799) from the southern North Sea (Reumer, Ten Broek & Galis, 2014). This was explained either as a direct result of a reduced gene pool due to population fragmentation towards its final extinction or as due to adverse conditions during early stages of pregnancy.

Our aim here is to test whether there are indications for similar conditions in another megaherbivore member of the mammoth steppe fauna: the woolly rhinoceros *Coelodonta antiquitatis* (Blumenbach, 1799). To this end, we investigated developmental abnormalities in the neck vertebrae, specifically the presence of cervical ribs. We checked the extensive collection of Late Pleistocene *C. antiquitatis* material in the Naturalis Biodiversity Centre (Leiden, the Netherlands) to estimate the incidence of cervical ribs. Additionally, we compared the resulting incidence of cervical ribs with those that we found in skeletons of all five extant African and Asian species of rhinoceros (*Dicerorhinus sumatrensis, Ceratotherium simum, Rhinoceros unicornis, R. sondaicus, Diceros bicornis*).

## Methods

### Specimens

We analyzed 32 seventh cervical vertebrae (C7) of the Late Pleistocene woolly rhino (*C. antiquitatis*) from the collection of the Naturalis Biodiversity Center (Naturalis) (Table 1). In order to identify diagnostic features of cervical and thoracal vertebrae of rhinoceroses, we analysed 56 complete skeletons of extant Rhinoceratidae, eight *Dicerorhinus sumatrensis*, 14 *Ceratotherium simum*, eight *Rhinoceros unicornis*, six *R. sondaicus*, twenty *Diceros bicornis* from seven collections: Naturalis Biodiversity Center, Leiden (Naturalis), the American Museum of Natural History, New York (AMNH), Muséum National d’Histoire Naturelle, Paris (MNHN), the Zoological Museum, University of Copenhagen (ZMUC), Field Museum of Natural History, Chicago (FMNH), Naturhistorisches Museum Wien, Viena (NHMW) and the Swedish Museum of Natural History, Stockholm (NRM) (see Table 2 for details).

**Table 1:** List of investigated specimens of *Coelodonta antiquitatis* (Late Pleistocene). All specimens (*n* = 32) come from The Netherlands. The presence of articulation facets of ribs was indicated anteriorly on the seventh cervical vertebra. All specimens are curated at Naturalis Biodiversity Center.

Collection no.	Age	Identity	Location
RGM 123268	Adult	C7	North Sea, Bruine Bank, 52 45N, 2 40E
RGM 123886	Adult	C7	North Sea, 30–40 miles WNW of IJmuiden
RGM 124895	Adult	C7	North Sea, 52 27N, 2 25E
RGM 132637	Adult	C7	North Sea, Bruine Bank, 52 40N, 2 55E
RGM 133133	Adult	C7	North Sea, South of Bruine Bank
RGM 138907	N/A	C7	North Sea, Bruine Bank, 52 30N, 3 00E
RGM 139898	Adult	C7	The Netherlands, Zuid-Willemsvaart, ’s-Hertogenbosch, prov. N. Brabant
RGM 146833	Adult	C/T	North Sea, 52 30N, 2 30E
RGM 152631	Adult	C7	North Sea, Bruine Bank, 52 30N, 3 00E
RGM 152709	Adult	C7	North Sea, Bruine Bank, 52 37N, 3 02E
RGM 153522	Adult	C7	North Sea, Bruine Bank, 52 10N, 2 50E
RGM 171473	Adult	C7	The Netherlands, Schaar van Colijnsplaat, prov. Zeeland
RGM 171525	Adult	C7	The Netherlands, Schaar van Colijnsplaat, prov. Zeeland, 51 36N, 3 52E
RGM 171542	Adult	C7	The Netherlands, Schaar van Colijnsplaat, prov. Zeeland
RGM 369367	Juvenile	C7	North Sea, South of Bruine Bank
RGM 369657	Adult	C7	North Sea, Bruine Bank, between 52 50 and 53 00N, 2 50 and 3 00E
RGM 388048	Adult	C7	North Sea, Bruine Bank, between 52 50 and 53 00N, 2 50 and 3 00E
RGM 388049	Juvenile	C7	North Sea, Bruine Bank, between 52 30 and 53 00N, 2 50 and 3 00E
RGM 400927	Juvenile	C7	North Sea, Bruine Bank, between 52 30 and 53 00, 2 50 and 3 00E
RGM 445933	Adult	C/T	The Netherlands, Westerschelde, Ellewoutsdijk, prov. Zeeland
RGM 445939	Adult	C7	The Netherlands, Ellewoutsdijk, Westerschelde, prov. Zeeland
RGM 55335	Juvenile	C7	The Netherlands, Westerschelde, Plaat van Baarland, prov. Zeeland
RGM 58226	Juvenile	C7	The Netherlands, Westerschelde, prov. Zeeland
RGM 63324	Juvenile	C7	The Netherlands, Hollands Diep, east of Moerdijkbrug; prov. N. Brabant
RGM 92687	Adult	C7	The Netherlands, ’s-Hertogenbosch, between Zuid-Willemsvaart and Aa, prov. N. Brabant
RGM 93342	Adult	C7	The Netherlands, Ellewoutsdijk, Westerschelde, prov. Zeeland
RGM 93408	Juvenile	C7	The Netherlands, Ellewoutsdijk, Westerschelde, prov. Zeeland
RGM 93449	Adult	C7	The Netherlands, Ellewoutsdijk, Westerschelde, prov. Zeeland
RGM 93473	Juvenile	C7	The Netherlands, Ellewoutsdijk, Westerschelde, prov. Zeeland
RGM 93477	Adult	C/T	The Netherlands, Westerschelde, Ellewoutsdijk, prov. Zeeland
RGM 93790	Adult	C/T	The Netherlands, between Zuid Willemsvaart en AA, ‘s Hertogenbosch, prov. N. Brabant
RGM 94549	Juvenile	C/T	The Netherlands, Westerschelde, Ellewoutsdijk, prov. Zeeland

**Table 2:** List of investigated seventh cervical vertebrae (C7) of extant Rhinocerotidae. All specimens (*n* = 59) are either wild-caught or collected in the field.

Species	Institute	Collection no.	Sex and age	Locality & remarks
***Dicerorhinus sumatrensis*** (G. Fisher, 1814)	Naturalis	RGM cat a	F, adult	Indonesia, Sumatra,Padang besi
		RGM cat b	M, adult	Indonesia, Sumatra
		RGM cat g	M, adult	Indonesia, Sumatra
	MNHN	MNHN-ZM-AC-1887-932	Adult	Indonesia, Sumatra
	ZMUC	ZMUC CN3791	F, adult	Indonesia, Sumatra
	AMNH	AMNH 54763	Juvenile	Burma; foramen transversarium at right side
		AMNH 54764	M, Juvenile	Burma
		AMNH 81892	Adult	Malaysia
***Ceratotherium simum*** (Burchell, 1817)	NRM	NRM 592359	n.a.	South Africa, Zululand
	NMW	NMW 3086	Adult	Sudan, Lado, 5°10N/31°32E, Equatoria Prov.
	MNHN	MNHN-ZM-AC-A7968	Adult	South Africa, Cape of Good Hope
	ZMUC	ZMUC CN2662	M, adult	Sudan, Joknyang forest, near Jur river
	FMNH	FMNH 29174	M, adult	Uganda, White Nile District, Rhino Camp
		FMNH 125413	M, adult	South Africa, Natal Province, 13km from Mkuze, Zululand District
	AMNH	AMNH 51855	Adult	Zaire
		AMNH 51856	Adult	Zaire
		AMNH 51857	Adult	Zaire
		AMNH 51858	Adult	Zaire
		AMNH 51859	Adult	Zaire
		AMNH 51860	Adult	Zaire
		AMNH 51861	Adult	Zaire
		AMNH 51862	Juvenile	Zaire
***Rhinoceros unicornis*** Linnaeus, 1758	MNHN	MNHN-ZM-AC-1960-59	Adult	n.a.
		MNHN 1792	Adult	n.a.
		MNHN-ZM-AC-1967-101	Adult	n.a.a
	AMNH	AMNH 35759	Adult	n.a.
		AMNH 54454	Adult	India
		AMNH 54456	Adult	India
		AMNH 119475	Juvenile	n.a.
	FMNH	FMNH 57639	M, adult	n.a.
***Rhinoceros sondaicus*** Desmarest, 1822	Naturalis	RGM cat a	M, adult	Indonesia, Java
		RGM cat c	M, adult	Indonesia, Java
		RGM ZMA 507	n.a.	Indonesia, Java
	MNHN	MNHN-ZM-AC-A7970	Adult	n.a.
		MNHN-ZM-AC-A7971	Juvenile	Indonesia, Java
	ZMUC	ZMUC CN 26	Adult	Indonesia, Java
***Diceros bicornis*** (Linnaeus, 1758)	Naturalis	RGM cat a	M, adult	South Africa, Cape of Good Hope
		RGM ZMA 506	Adult	n.a.
		RGM 5738	M, Adult	n.a.
	MNHN	MNHN-ZM-AC-1941-208	M, juvenile	n.a.
		MNHN-ZM-AC-1944-278	M, adult	n.a.
		MNHN-ZM-AC-1936-644	F, adult	n.a.
		MNHN-ZM-AC-A7969	Adult	South Africa, Cape of Good Hope
	ZMUC	ZMUC CN36..	Adult	Ethiopia, Abessnie
		ZMUC CN 3653	M, adult	Africa
		ZMUC CN 4435	F, adult	Kenya, Machanga
	AMNH	AMNH 14136	Adult	n.a.
		AMNH 27757	Juvenile	Zaire
		AMNH 34739	Adult	Kenya
		AMNH 35319	Juvenile	n.a.
		AMNH 81805	Juvenile	South Africa
		AMNH 113776	Adult	Zaire
		AMNH 113777	Adult	n.a.
		AMNH 245690	Adult	n.a.
	FMNH	FMNH 57809	M, adult	Africa, Oideani Mountains
		FMNH 127848	F, adult	Tanzania

**Notes.**

F female M male n.a. not available

All included woolly rhino specimens are of Late Pleistocene age and originate from the North Sea. No absolute datings are available due to the preservational condition of the specimens. Only locality and sometimes stratigraphic data are available, from which the geological age can be roughly estimated (Table 1). All our specimens were retrieved from either the North Sea (area of the Bruine Bank), the Dutch Schelde deltaic region (province Zeeland) or somewhat more inland (the Netherlands, province Noord Brabant). The Bruine Bank (Brown Ridge in English) is a sandbank constituting an elevation in the Southern Bight and consisting of alluvial sediments. The Schaar van Colijnsplaat is a marine channel in the river Oosterschelde (Eastern Scheldt). Ellewoutsdijk is a dike of the river Westerschelde (Western Scheldt). Plaat van Baarland is a tidal flat in the Westerschelde. All inland localities (Zuid-Willemsvaart, Overijsselse Vecht, Hollands Diep) are waterways. The southern bight of the North Sea consisted of ice-free dry land during the Weichselian (115,000–12,600 BP; known as the Würm glaciation in Europe’s Alpine region and the Devensian glaciation in North America), with three or four intervals with marine transgressions during less cold phases (Post, 2005). Our specimens cannot be dated more precisely than Weichselian; that is, between approximately 115,000 BP and 36,000 BP, the latter date being the last occurrence of this species in the west (Stuart & Lister, 2012), except for the specimens from the Bruine Bank, which are likely not older than c. 60,000 years (Mol et al., 2006). All specimens of extant Rhinocerotidae were wild-born and as far as dates are available, the majority lived in the early 20th century or earlier. 47 out of 56 died in the wild as a result of zoological collecting activities and nine lived after capture in zoos and died there.

### Cervico-thoracic transitional vertebrae

We analyzed the C7 vertebrae for the presence or absence of articulation facets of cervical ribs. We follow a conservative approach here by assuming that the ribs, if present, are cervical ribs and not rudimentary first ribs, because the incidence of cervicalized thoracic vertebrae in mammals is extremely low. In a meta-analysis of deceased human foetusses and infants, Galis et al. (2006) and Ten Broek et al. (2012) found that respectively 0.98% (9.8% erroneously in the former article) and 1.3% of all C/T transformations consisted of rudimentary first ribs (of which half unilateral) against ∼99% cases of cervical ribs. Bradley (1901) describes such a transitional T1 in a horse, and he as well remarks that the condition is extremely rare. The extra-ordinary low incidence of rudimentary first ribs does in our view not support such an interpretation for cervico-thoracic transitional vertebra in the woolly rhinoceros. In addition, a somewhat cervicalized first thoracic vertebra (albeit not involving a change in the ribs) with a cervical type of zygapophyses, or atypical cervico-thoracic junction, as found in the extant giraffe (*Giraffa camelopardalis*) coincides with homogenization of C3–C7 and an extremely long neck (Danowitz & Solounias, 2015). This condition is not present in the woolly rhino, where C3–C7 each have their own distinctive identity.

**Figure 1:**
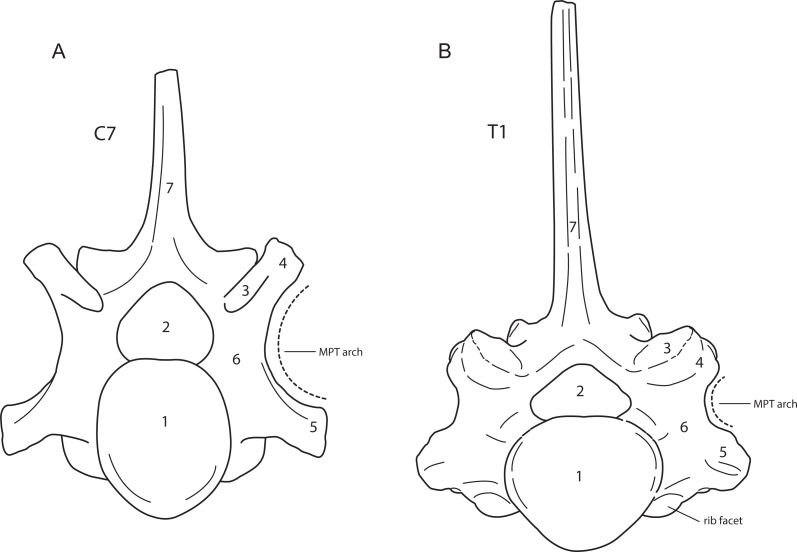
Anterior view of a generalised seventh cervical vertebra (A) and first thoracic vertebra (B) of the woolly rhinoceros. 1, anterior articular surface; 2, vertebral foramen or vertebral canal; 3, prezygapophysis or anterior articular process; 4, mammillary process; 5, transverse process; 6, pedicle; 7, neural or dorsal spine, MPT arch = arch formed by the mammillary process, the pedicle and the transverse process. Note that the cervical vertebra does not bear a rib facet on the vertebral body. Illustration: Erik-Jan Bosch.

**Figure 2:**
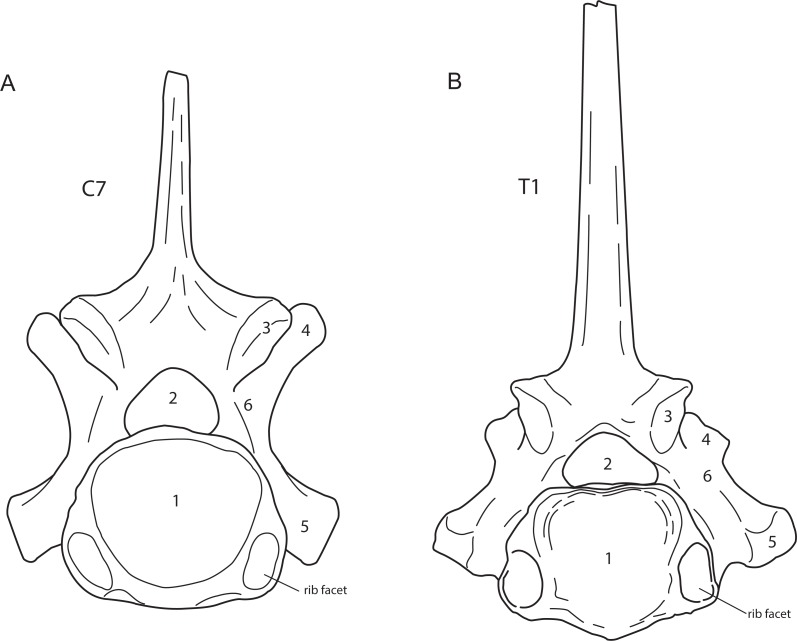
Posterior view of a generalised seventh cervical vertebra (A) and first thoracic vertebra (B) of the woolly rhinoceros. 1, posterior articular surface; 2, vertebral foramen or vertebral canal; 3, postzygapophysis; 4, prezygapophysis; 5, transverse process; 6, pedicle. Note that both vertebrae bear a rib facet bordering the posterior articulation. Illustration: Erik-Jan Bosch.

**Figure 3:**
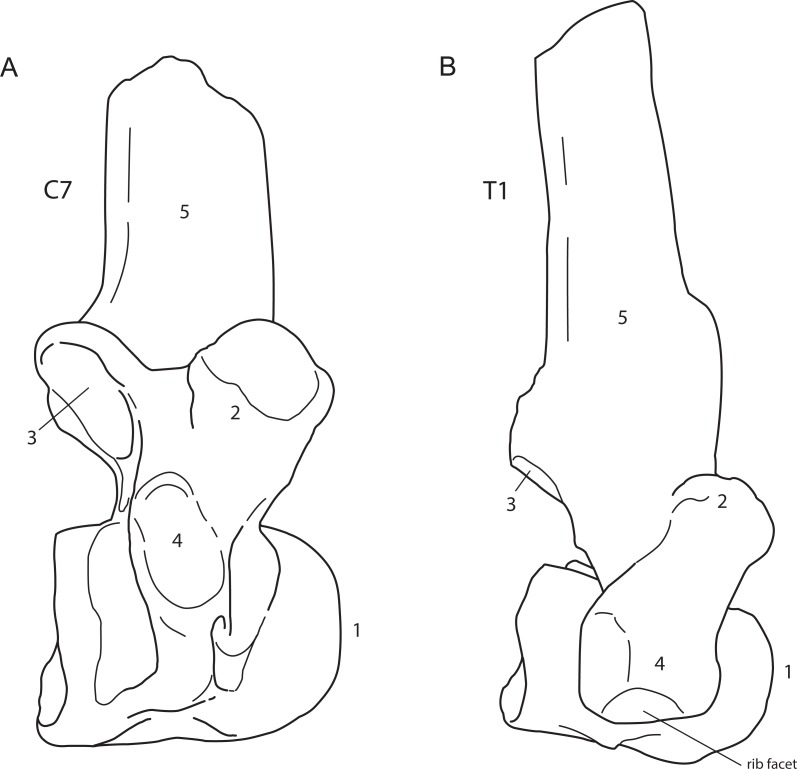
Lateral view of a generalised seventh cervical vertebra (A) and first thoracic vertebra (B) of the woolly rhinoceros. Lateral view of a generalised seventh cervical vertebra (A) and first thoracic vertebra (B) of the woolly rhinoceros. 1, anterior articulation; 2, prezygapophysis; 3, postzygapophysis with articular area indicated; 4, transverse process; 5, dorsal or neural spine. Note that the cervical vertebra does not bear a rib facet on the transverse process. Illustration: Erik-Jan Bosch.

The presence of cervical ribs can be deduced from articulation facets on the anterior side of the centrum and the transverse processes of C7 (Fig. 1). To distinguish a transitional cervico-thoracic (C/T) vertebra with ribs from a first thoracic vertebra (T1), we scored 12 discrete characters diagnostic for either C7 or T1 (Figs. 1–3), amongst others shape parameters of the vertebra and size of the rib facets (Table 3), based on Borsuk-Białynicka (1973), Protero (2005), Diedrich (2006) and our own observations on 56 complete skeletons of extant rhinoceroses. When a vertebra had rib facets and six or more cervical or cervico-thoracic transitional characters, thus 50% cervical in morphology, we considered the vertebra to have a transitional C/T identity, whereas a vertebra with rib facets but fewer cervical or cervico-thoracic transitional characters, we considered it to be a regular T1. An additional diagnostic character considered here is that C/T vertebrae often display left–right asymmetry in the extent of the homeotic transformation, for instance differences in the size of the left and right ribs (Varela-Lasheras et al., 2011; Ten Broek et al., 2012).

**Table 3:** List of diagnostic characters. Features of cervical and thoracic vertebral elements used in this study to identify a vertebra as either C7, T1 or intermediate.

**Feature**		**View**	**C7**	**T1**
**Centrum**				
a	Shape of anterior articular face	Rostral	Oval—higher than wide, with dorsal border convex	Heart-shaped—at least as wide as high, with approx. straight dorsal border
b	Shape of posterior end (adult)	Caudal	Dorsal width clearly exceeds ventral width, may have dimple midway dorsally, ventrally convex	Squarish, dorsal width approx equals ventral width, ventrally straight or tapering
	Shape of posterior end (juvenile)	Caudal	Wider than high (at least 69%), Ventrally rounded, Shallow dimple midway dorsally and indistinct trace of rib facets	Wider than high (less than 64%), ventrally tapering, sharp dimple midway dorsally and prominent impression of rib facets
c	Ventral keel	Ventral	Single or bilateral median tubercle, strongly developed	No tubercles, may be (very) weakly developed
d	Shape of anterior articular face	Ventral	Deep and rounded, A–P length approx. 40–50% of the width of centrum	Shallow and flat, A–P length approx. 25–35% of width of centrum
**Vertebral foramen**				
e	Shape	Rostral	Wide (large), H/W ratio 75% or more	Narrow (small), H/W ratio 73% or smaller
**Prezygapophyse**				
f	Left-right distance	Rostral	Large—shortest distance approx. 50% of total width at same level	Small—shortest distance approx. 35% of total width at same level
g	Position relative to transverse process	Dorsal	More anterior than transverse process	Overhangs transverse process
**Postzygapophyse**				
h	Articular facet	Lateral	Visible for greatest part (latero-caudally directed)	At most minimally visible (caudally directed)
i	Articular facets	Caudal	Widely spaced (inner side lateral of neural spine)	Narrow spaced (inner side close to centre of neural spine)
**Mammilary process, pedicle and transverse process**				
j	Outer arch formed by mammilary process, pedicle and transverse process	Rostral	Long—at least 85% of height of centrum	Short—at most 70% of height of centrum
**Rib facets**				
k	At transverse process	Lateral	Absent	Present (large)
l	At centrum	Rostral	Absent	Present (large)

### Statistical tests

To compare the prevalence of cervical rib facets between woolly rhinos and extant rhinocerotid species we used a Fisher’s exact test, which is suitable for small sample sizes (*n* = 32 and *n* = 56) (McDonald, 2014). *P*-values < 0.05 are here considered as significant.

## Results

**Figure 4:**
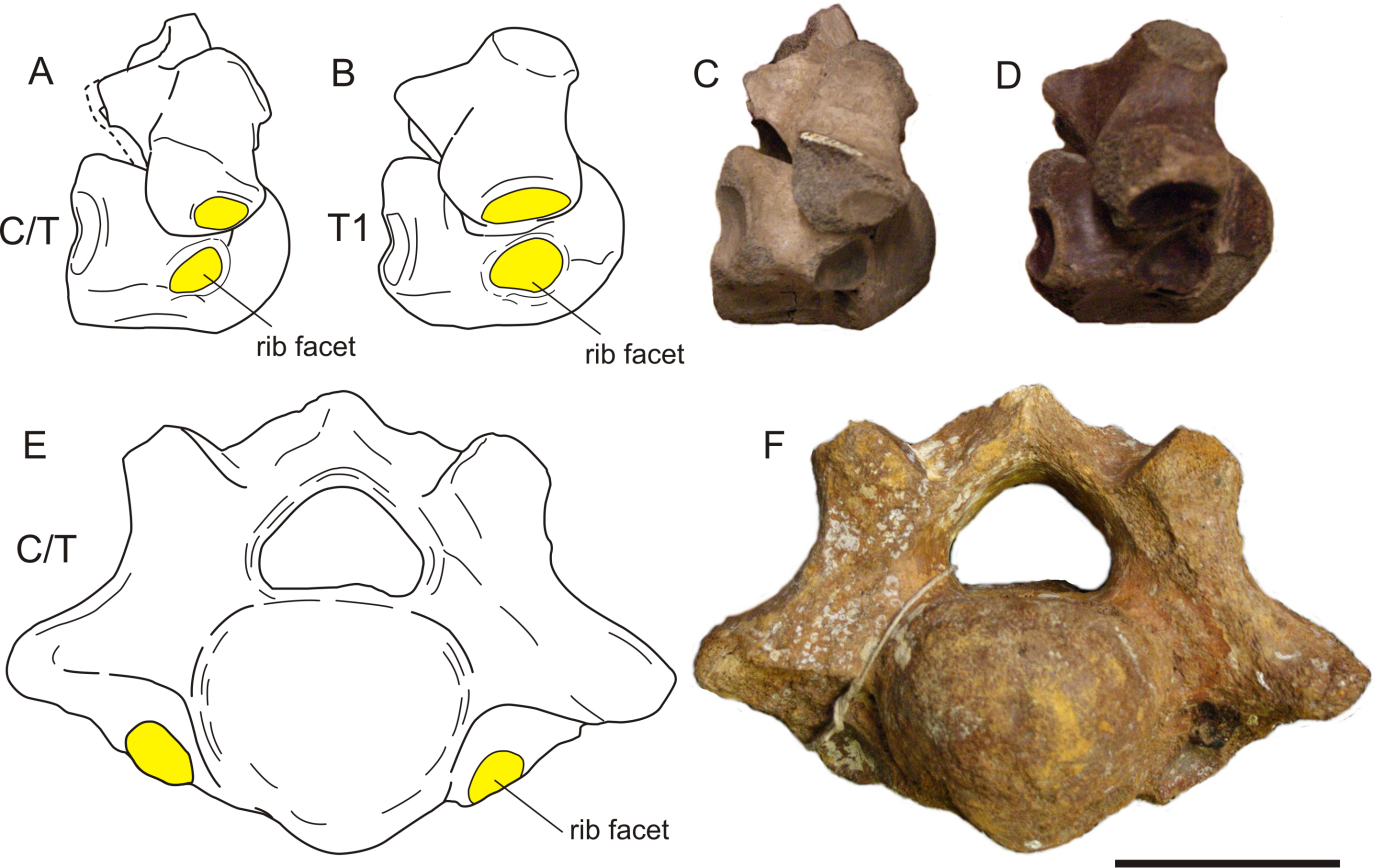
Two cervico-thoracic vertebrae of the woolly rhinoceros with small rib facets. (A) Schematic drawing of a C/T in lateral view compared to a normal T1 (B), showing the much smaller size of the rib facets in C/T than in T1. (C–D) Photographs of the same specimens (A–B). (E) Schematic drawing of a C/T with anterior rib facets of unequal size and position. Rib facets are indicated in yellow. (F) Photograph of the same specimen (E). Specimens RGM 93790 (A, C), RGM 97393 (B, D), RGM 146833 (E, F). For transitional features, see Table 4. Illustration: Erik-Jan Bosch and George Lyras.

**Figure 5:**
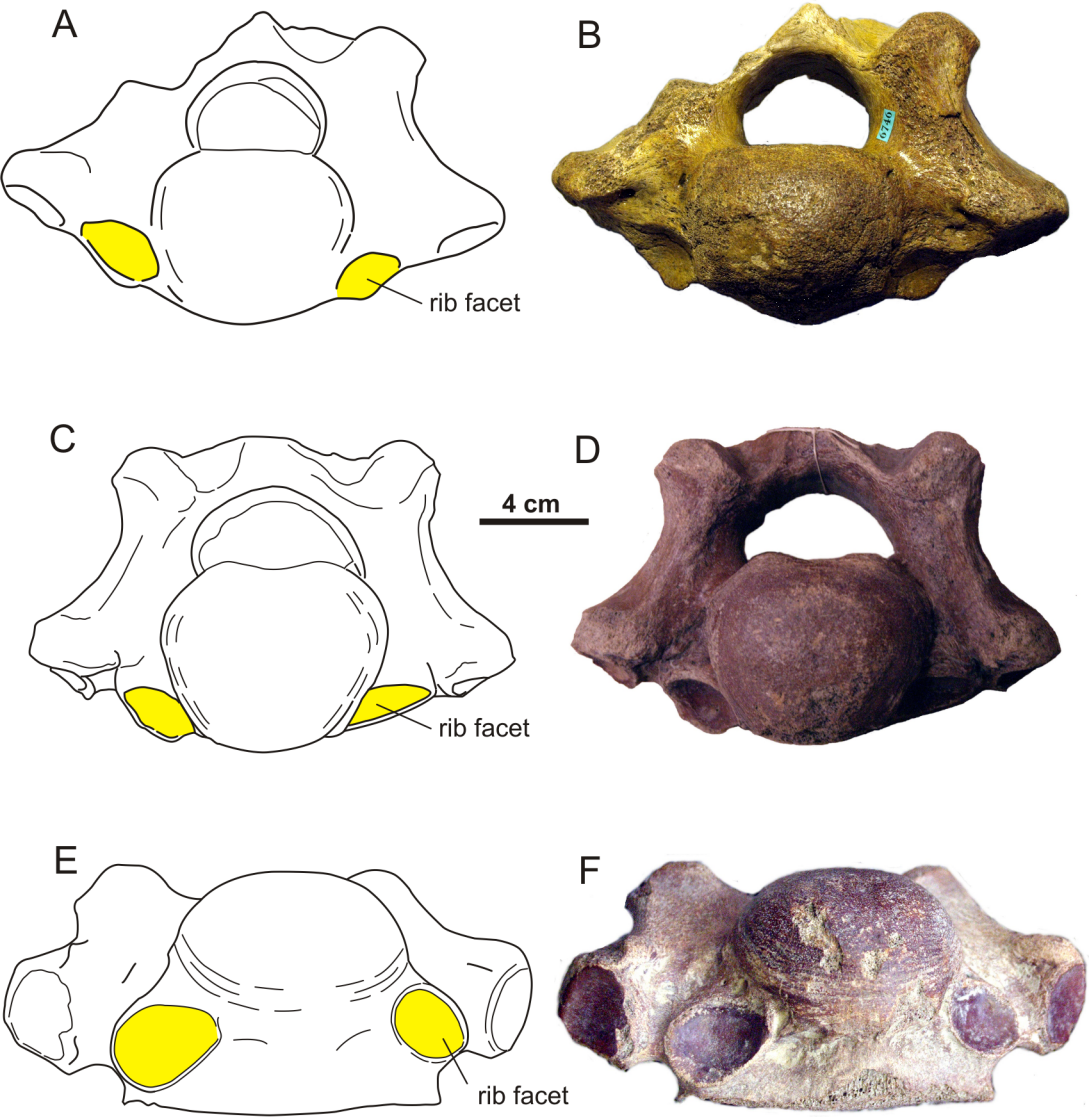
Asymmetrical transitional cervico-thoracic vertebrae of the woolly rhinoceros (*Coelodonta antiquitatis*). These three vertebrae are asymmetrical in the size as well as the position of the rib facet. (A–B) Schematic drawing and photograph of RGM 445933 (anterior view). (C–D) Schematic drawing and photograph of RGM 93477 (anterior view). (E–F) Schematic drawing and photograph of RGM 94549 (ventral view). For transitional features of A–F, see Table 4. Illustration: Erik-Jan Bosch and George Lyras.

**Figure 6:**
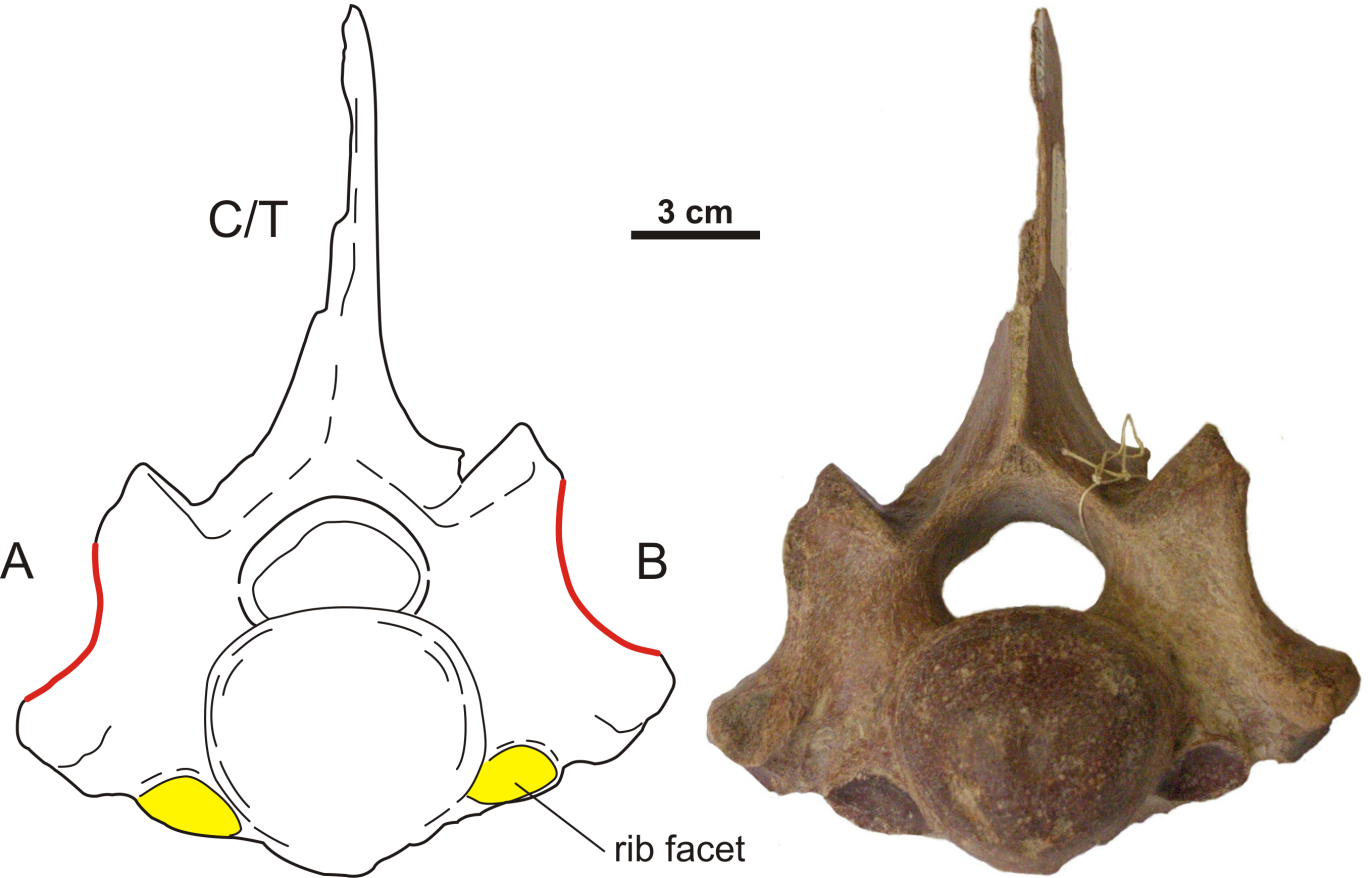
Anterior view of an asymmetrical transitional cervico-thoracic vertebra of the woolly rhinoceros. The right transverse process (A) is placed more ventrally and bears a larger rib facet than the left side process (B). The arch formed by the transverse process, the pedicle and the mammilary process (MPT arch) at the left side (B) resembles that of C7 (cervical), but of T1 (thoracic) at the right side (A), resulting in a somewhat distorted shape. Additionally, the prezygapophysis is placed more ventrally at the right side (A) than at the left side (B). Specimen RGM 94549. For transitional features of this specimen, see Table 4. Photo credit: George Lyras.

We found five transitional C/T vertebrae, 24 normal C7 vertebrae, and three C7 vertebrae with one or two transitional characters for *C. antiquitatis* (Figs. 4–6, Tables 1 and 4). The incidence of transitional C/T vertebrae (15.6%) is significantly higher than for the extant Rhinocerotidae where we found no transitional C/T vertebrae and 56 normal C7 vertebrae (*p* = 0.005) (Table 2). Judging from the rib facets, the cervical ribs were quite large and none of them was fused to the transverse process as is usually the case for small cervical ribs (Cave, 1975; Varela-Lasheras et al., 2011). Two of the five C/T vertebrae show small anterior rib facets relative to those of a T1 (RGM 93477, RGM146833) (Fig. 4), and three of the five C/T vertebrae displayed a conspicuous asymmetry in the size of the rib facets and other shape parameters (RGM 94549, RGM 445933, RGM 93790) (Fig. 5). The most prominently asymmetric specimen is RGM 94549, where the left anterior rib facet is considerably smaller and lies more dorsally than the one on the right and the transverse process and mammilary process are asymmetrically placed (Fig. 6).

## Discussion

The incidence of transitional C/T vertebrae in our set of Late Pleistocene *C. antiquitatis* from the North Sea and the Netherlands is high (15.6%, *n* = 32) and significantly higher than that of extant rhinocerotids (0%, *n* = 56). In humans, the incidence in the general population is estimated to be between 0.5 and 1% and is only higher than 1% in diseased or isolated populations (Galis et al., 2006). A particularly high incidence has been found in children with cancer (15–25%; Schumacher, Mai & Gutjahr, 1992; Merks et al., 2005; Galis & Metz, 2003) and deceased fetuses (approximately 50%; Galis et al., 2006; Furtado et al., 2011; Ten Broek et al., 2012; Schut et al., 2016). Investigations of skeletons of other extant mammalian species found support for an association between cervical ribs (C/T vertebrae) and defects, such as fused vertebrae and ossification abnormalities (Varela-Lasheras et al., 2011; Ten Broek et al., 2012). This applies inter alia also to rudimentary first ribs, which were in all cases coupled to multiple major abnormalities (Galis et al., 2006). Late Pleistocene woolly mammoths from the North Sea display an unusually high incidence of cervical ribs, much higher than that of extant elephants (Reumer, Ten Broek & Galis, 2014). Interestingly, both woolly rhinos and mammoths have quite large cervical rib articulation facets, indicating cervical ribs that are substantially larger than usually found in humans and other extant mammals (see Cave, 1975; Bots et al., 2011; Varela-Lasheras et al., 2011; Ten Broek et al., 2012; Reumer, Ten Broek & Galis, 2014 for examples), though not as large as first ribs. Size of cervical ribs was found to be negatively correlated with fitness in transgenic mice (Jeannotte et al., 1993; see also Bots et al., 2011). Importantly, there are a few cases of woolly rhinos with developmental abnormalities. An isolated deciduous molar of a woolly rhino from Italy (Grotta di Fumane, Verona) retrieved from a Palaeolithic layer dated between 37,000 and 32,000 years ago (*C*^14^; Bartolomei et al., 1994) shows an enamel defect (hypoplastic form of amelogenesis imperfecta) (Billia & Graovac, 1998). Perhaps not coincidentally, the geological age coincides with the last occurrences of this species in the west (Stuart & Lister, 2012). In humans, such a structural enamel anomaly is hereditary (Stephanopoulos, Garefalaki & Lyroudia, 2005). Also, there are three reported cases of supernumerary teeth (hyperodontia) in *C. antiquitatis* (4th upper molar, 3rd lower molar in Garrutt, 1990; 3rd lower molar in Hillman-Smith et al., 1986). In humans, supernumerary teeth are commonly seen with several congenital genetic disorders (Subasioglu et al., 2015). One of the woolly rhino specimens reported by Garrutt (1990) also has an osteoma (a benign bone tumor) at the top of the occipital bone (Garrut, 1994), which might indicate a wider spectrum of abnormalities in this specimen.

The exceptionally high incidence of large cervical ribs in Late Pleistocene woolly rhinos and mammoths can be due to two factors. Firstly, it can be due to a high rate of inbreeding in declining populations potentially resulting in drift and preservation of less favorable genetic variants. A large frequency of cervical ribs (7.5%) in adults has been observed in an isolated human population in Sicily (Palma & Carini, 1990), in inbred pedigreed dogs (11.4% Breit & Kunzel, 1998) and in inbred minipigs (11%, Jorgensen, 1998). Generally, in inbred mammals there is an increased incidence of congenital anomalies (Cristescu et al., 2009; Räikkönen et al., 2013). Recent studies have shown that the genetic diversity was indeed extremely low in Late Pleistocene mammoths in Siberia (Miller et al., 2008; Nyström et al., 2012). In woolly rhinoceroses, unfortunately no research has been carried out on genetic diversification. An indirect indication that such a loss of genetic variation might have existed is that the range of the woolly rhinoceros gradually disintegrated into isolated spots before their extinction towards the end of the Late Pleistocene (Markova et al., 2013).

**Figure 7:**
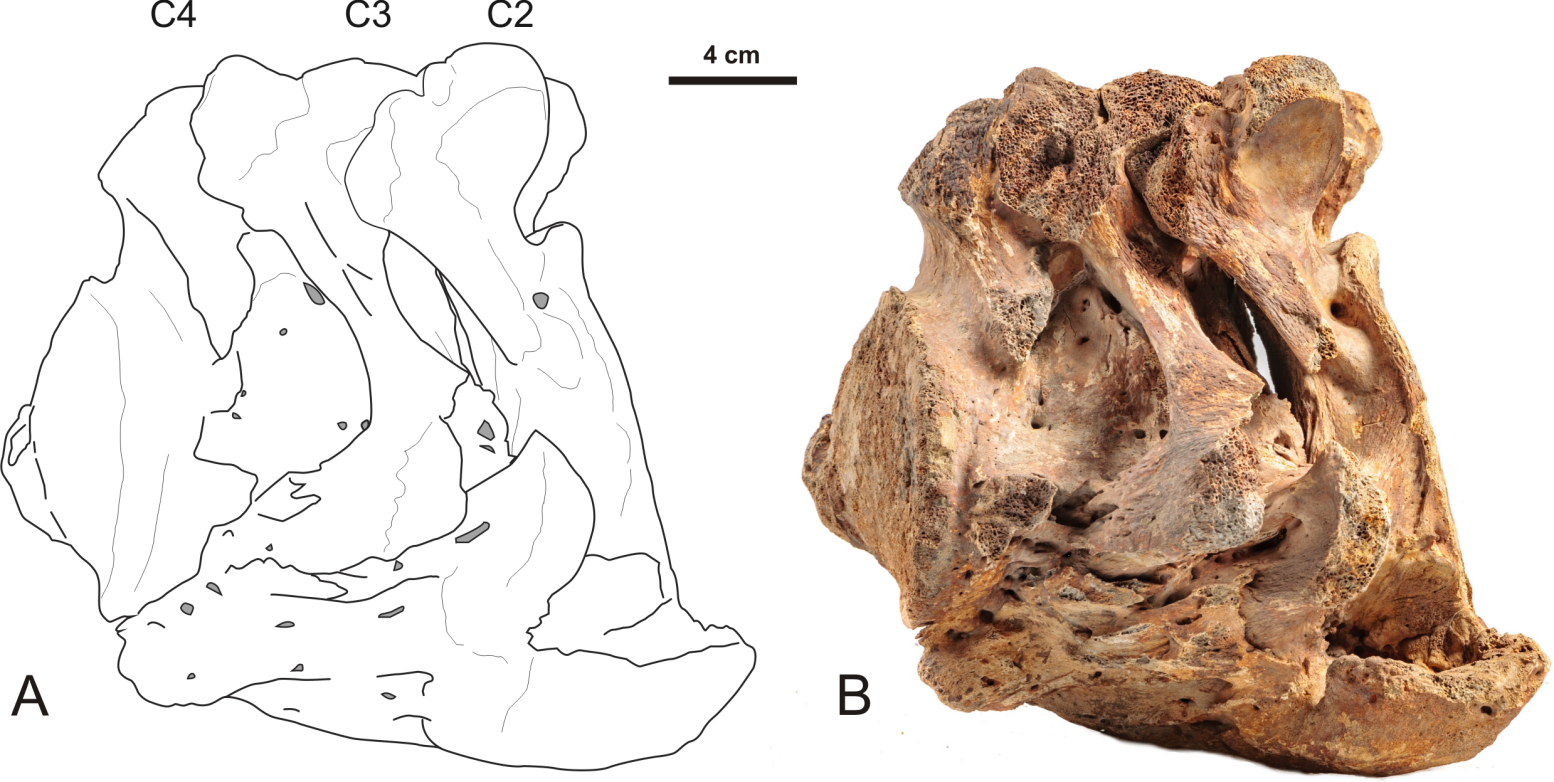
Lateral view of a partially fused 2nd, 3rd and 4th cervical vertebra of a Late Pleistocene woolly mammoth from the North Sea with bone overgrowth originating from the intervertebral disk. (A) Schematic drawing, indicating the second, third and fourth cervical vertebra. (B) Photograph of the same specimen. Partial fusion can have many causes, amongst others as a reaction to severe spondylosis (degeneration of the intervertebral disks and vertebral bodies) or infectious spondylo-osteomyelitis in case a bacterial infection is the underlying cause. Bone spurs (osteophytes or syndesmophytes) may arise primarily along the ventral and lateral intervertebral joint margin and eventually merge to form a firm bony bridge between the vertebrae, resulting in total immobility of the joint (ankylosis) and sometimes pressure on blood vessels and nerves. The bone spurs may infiltrate adjoining tissue and thus form a larger mass than just a bony bridge. The overgrowth observed in this specimen may also represent a cancerous process such as an osteoma that has been regularly observed in other Late Pleistocene woolly mammoths (Leshchinskiy, 2012; Leshchinskiy, 2015). Specimen RGM 153025. Photo credit: Joris van Alphen, http://portfolio.jorisvanalphen.com/.

**Figure 8:**
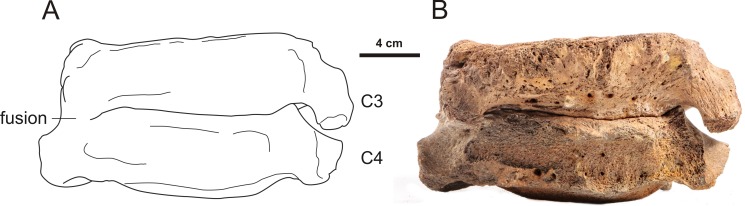
Lateral view of a 3rd and 4th cervical vertebra of the woolly mammoth (Late Pleistocene, North Sea). (A) Schematic drawing indicating the individual vertebrae and the region of fusion. (B) Photograph of the same specimen. This specimen shows a segmentation defect, resulting in a partial fusion. Specimen RGM 400795. Photo credit: Joris van Alphen, http://portfolio.jorisvanalphen.com/.

**Figure 9:**
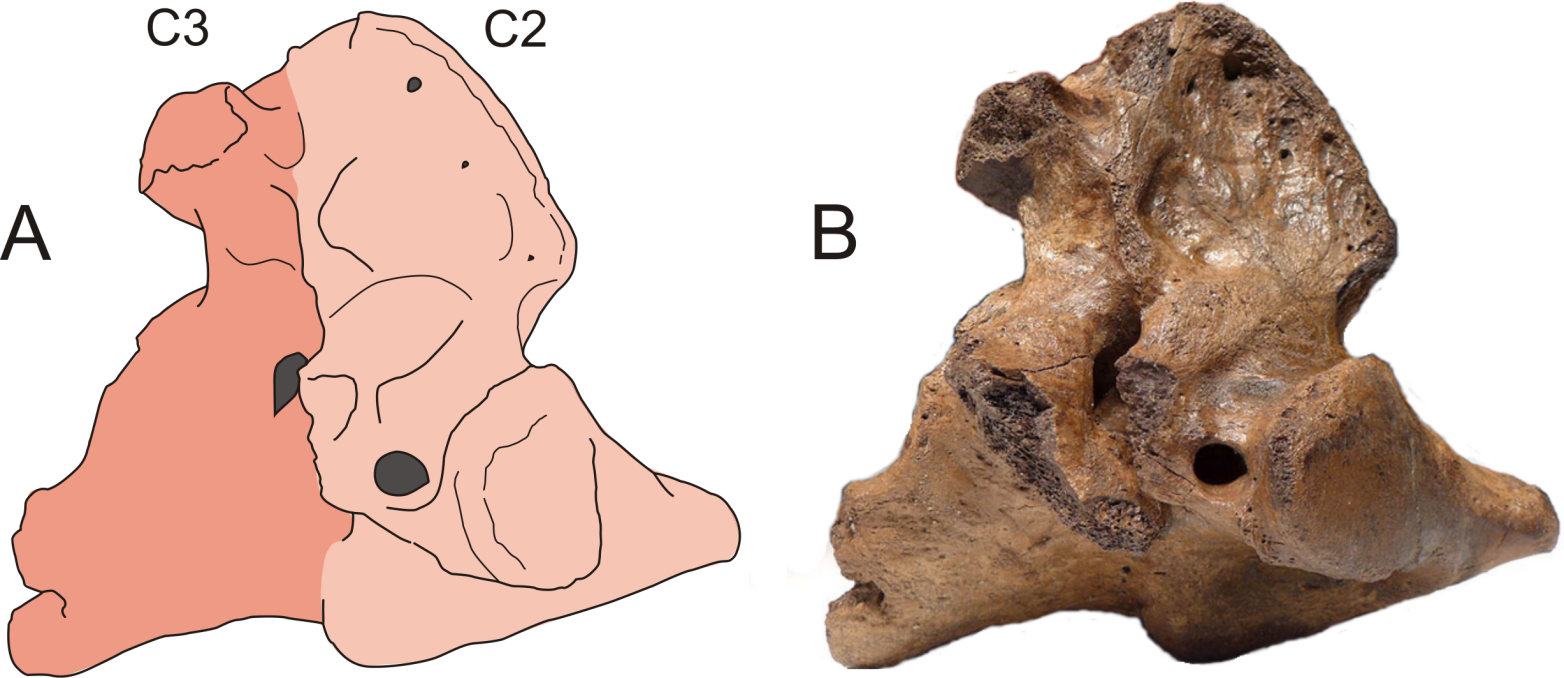
An almost completely fused second and third cervical vertebra of the woolly rhinoceros (*Coelodonta antiquitatis*). (A) Schematic drawing indicating the individual second and third cervical vertebra. (B) Photograph of the same specimen (lateral view, adult; length 190.5 mm, height 177.8 mm). The specimen originates from Central Europe, dating approx. 80,000–20,000 years ago. The fusion is likely due to a segmentation defect, due to disturbances of the early embryonic segmentation process of the prevertebrae (somites). The contact area between the two elements does not show any sign of osteophyte formation, which is typical for fusions due to reactive bone formation (osteophytes) in degenerative processes. Specimen LMX110. Photo credit: Bryon McWilliams, PaleoDirect, http://www.PaleoDirect.com.

A second cause of the increased incidence of cervical ribs may be harsh conditions that impact early pregnancies, because diseases, famine, cold and other stressors can lead to disturbances of early development, that can result in the induction of cervical ribs (e.g., Sawin, 1937; Li & Shiota, 2000; Wéry et al., 2003; Chernoff & Rogers, 2004; Steigenga et al., 2006). Harsh conditions during the Late Pleistocene, a period of intense climatic fluctuations and ecosystem instability, are plausible (Brace et al., 2012). The extinction of the woolly mammoth and the woolly rhinoceros has been related to the Late Glacial interstadial warming event and increased precipitation, especially snowfall, together with a replacement of forbs (non-graminoid herbaceous vascular plants) and mosses—the typical diet of woolly mammoths and rhinos according to (Willerslev et al., 2014)—by less nutritious shrubs and trees and an increase in the amount of fibrous grasses with low nutritional value (Stuart & Lister, 2012). The high incidence of bone dystrophy and severe arthrosis found in young mammoths of Northern Eurasian Late Pleistocene populations are assumed to be caused by mineral deficiencies in pregnant females and developing calves, especially in the geologically youngest populations (Leshchinskiy, 2012; Leshchinskiy, 2015). Spondylosis (a collective term for degenerative conditions affecting the disks, vertebral bodies, and/or associated joints; Middleton & Fish, 2009) also occurs and may lead to vertebral fusions (Fig. 7). Segmentation defects, due to disturbances of the early embryonic segmentation process of the prevertebrae (somites) can also cause fusions of vertebrae (Fig. 8). Such congenital defects are commonly associated with cervical ribs (Varela-Lasheras et al., 2011; Ten Broek et al., 2012). A perhaps similar segmentation defect in a woolly rhinoceros may be provided by a pathological fusion of a second and third vertebra (Fig. 9). As in the case of the woolly mammoth (Fig. 8), these abnormalities were not necessarily fatal but persisted into adulthood. In general, studies of abnormalities in extinct megaherbivores are scarce, other than incidental observations of abnormalities (see also the above-mentioned amelogenesis imperfecta and supernumerary teeth in woolly rhinoceroses), hampering any statistical evaluation of their occurrence or their relation to other developmental anomalies. At the present stage of our knowledge, a combination of inbreeding and harsh conditions may be the most likely explanation for the extremely high incidence of cervical ribs in two Ice Age megaherbivores, the woolly mammoths and rhinoceroses.

**Table 4:** Transitional vertebrae of the woolly rhinoceros. List of last cervical vertebrae and first thoracic vertebrae of the woolly rhinoceros (*Coelodonta antiquitatis*; Late Pleistocene, Netherlands) with at least one transitional character. For specification of character scores, see Table 3. Vertebrae with at least six transitional characters (highlighted in grey) are considered transitional C/T vertebrae.

Specimen	C7 features	C/T features	T1 features	Identity
RGM 139671	f, h	–	a–e, g, i–l	T1
RGM 146833	b, d, e	f, j, k, l	a, g	C/T
RGM 171525	a, b, e, g–l	d, f	–	C7
RGM 369367	a–e, g–l	f	–	C7
RGM 369657	a, b, e–l	d	–	C7
RGM 445933	b, d, e, g, j	f, l	a, k	C/T
RGM 55336	–	f	a–e, g, j–l	T1
RGM 93477	c, g	a, e, f, j	b, d, k, l	C/T
RGM 93479	f	–	a–e, g–l	T1
RGM 93485	e	–	a, d, f, g, j–l	T1
RGM 93790	e	a, c, d, f, j, k, l	b, g	C/T
RGM 94549	c, e, f, g, h, i	b, d, j, l	a, k	C/T

The relatively high incidence of cervical ribs might present a transitional stage in an evolution towards a shorter neck in the woolly rhinoceros lineage. This is, however, in our opinion unlikely for two reasons. First, there does not seem to be a selective advantage to a shorter neck, because this species likely carried a fat hump above the shoulders as inferred from the presence of extremely long dorsal spines of the anterior thoracic vertebrae (Kahlke, 1999), which is mechanistically incompatible with a shorter neck. Second, the evolution towards fewer neck vertebrae in mammals is restricted to the sloth *Choloepus* and manatee *Trichechus*. Both are extremely slow moving species with an extremely low metabolism. Both taxa experienced, therefore, relaxed selection with adverse side-effects of a changed number of cervical vertebrae not negatively affecting their fitness (Varela-Lasheras et al., 2011). This is unlikely to have been the case for the woolly rhinoceros, a species that had to be relatively fast and agile to escape or fight predators like wolves and hyenas.

Our study found no cervical ribs in the investigated extant Asian and African rhinos. This might erroneously lead us to the conclusion that rhinoceros populations today are thriving and healthy. However, we have to keep in mind that all analysed specimens date back to times when populations were indeed almost certainly thriving. With a few exceptions, they were captured before the mid-20th century. Especially the past few decades saw very severe declines in their numbers, for example, greater than 80% in 60 years for the Sumatran rhinoceros (Van Strien et al., 2008), and a loss of 69% of the mitochondrial genetic variation in the black rhinoceros (Moodley et al., 2017). Our data are thus based on a situation that differs essentially from today. Furthermore, there are indications that the level of genetic variation in extant rhinos is still high because of the very rapid severe population decline. Such is the case in the greater one-horned rhinoceros of Chitwan National Park of Nepal, and is likely due to a compression of surviving populations into the park area, thereby concentrating genetic variation (Dinerstein, 2003). Monitoring of the skeletal health of rhinos, including the presence of cervical ribs, would improve our power to assess their genetic status on the long term and predict their chances of survival.

## Conclusion

In conclusion, the high incidence and large size of the cervical ribs in the Late Pleistocene woolly rhinoceros of the North Sea indicates a strong vulnerability, given the association of cervical ribs with diseases and congenital abnormalities in mammals in general. If so, that vulnerable condition in woolly rhinoceroses and mammoths may well have contributed to their eventual extinction (Reumer, Ten Broek & Galis, 2014).

## Additional Information and Declarations

### Data Availability

The following information was supplied regarding data availability: The raw data is included in the tables and figures in the manuscript.

## References

[ref-1] Bartolomei G, Broglio A, Cassoli PF, Castelletti L, Cattani L, Cremaschi M, Giacobini G, Malerba G, Maspero M, Peresani M, Sartorelli A, Tagliacozzo A (1994). La Grotte de Fumane: un site aurignacien au pied des Alpes. Preististoria Alpina (Museo Tridentino di Scienze Naturali).

[ref-2] Bateson W (1894). Materials for the study of variation.

[ref-3] Billia EME, Graovac SM, Mayhall JT, Heikkinen T (1998). Amelogenesis Imperfecta on a deciduous molar of *Coelodonta antiquitatis* (Blumenbach) (Mammalia, Perissodactyla, Rhinocerotidae) from Grotta di Fumane (Verona, Northern Italy): a rare case report.

[ref-4] Borsuk-Białynicka M (1973). Studies on the Pleistocene Rhinoceros *Coelodonta antiquitatis* (Blumenbach). Palaeontologica Polonica.

[ref-5] Bots J, Wijnaendts LC, Delen S, Van Dongen S, Heikinheimo K, Galis F (2011). Analysis of cervical ribs in a series of human fetuses. Journal of Anatomy.

[ref-6] Brace S, Palkopoulou E, Dalèn L, Lister AM, Miller R, Otte M, Germonprè M, Blockley SP, Stewart JR, Barnes I (2012). Serial population extinctions in a small mammal indicate Late Pleistocene ecosystem instability. Proceedings of the National Academy of Sciences of the United States of America.

[ref-7] Bradley OC (1901). On a case of rudimentary first thoracic rib in a horse. Journal of Anatomy and Physiology.

[ref-8] Breit S, Kunzel W (1998). Osteologische Besonderheiten an Wirbelsaulen von Rassehunden: eine rontgenologische und morphologische Studie. Wiener Tierarztliche Monatsschrift.

[ref-9] Cave AJE (1975). The morphology of the mammalian cervical pleurapophysis. Journal of Zoology.

[ref-10] Chernoff N, Rogers JM (2004). Supernumerary ribs in developmental toxicity bioassays and in human populations: incidence and biological significance. Journal of Toxicology and Environmental Health, Part B.

[ref-11] Cristescu R, Cahill V, Sherwin WB, Handasyde K, Carlyon K, Whisson D, Herbert CA, Carlsson BLJ, Wilton AN, Cooper DW (2009). Inbreeding and testicular abnormalities in a bottlenecked population of koalas (*Phascolarctos cinereus*). Wildlife Research.

[ref-12] Danowitz M, Solounias N (2015). The cervical osteology of *Okapia johnstoni* and *Giraffa camelopardalis*. PLOS ONE.

[ref-13] Diedrich CG (2006). By ice age spotted Hyeanas removed, cracked, nibbled and chewed skeleton remains of *Coelodonta antiquitatis* (Blumenbach 1799) from the Lower Weichselian (Upper Pleistocene) freeland prey deposit site Bad Wildungen-Biedensteg (Hessia, NW Germany). Journal of Taphonomy.

[ref-14] Dinerstein E (2003). The return of the Unicorns: the natural history and conservation of the greater one-Horned Rhinoceros.

[ref-15] Furtado LV, Thaker HM, Erickson LK, Shirts BH, Opitz JM (2011). Cervical ribs are more prevalent in stillborn fetuses than in live-born infants and are strongly associated with fetal aneuploidy. Pediatric and Developmental Pathology.

[ref-16] Galis F (1999). Why do almost all mammals have seven cervical vertebrae? Developmental constraints, Hox genes, and cancer. Journal of Experimental Zoology.

[ref-17] Galis F, Metz JAJ (2003). Anti-cancer selection as a source of developmental and evolutionary constraints. BioEssays.

[ref-18] Galis F, Van Dooren TJ, Feuth JD, Metz JA, Witkam A, Ruinard S, Steigenga MJ, Wijnaendts LC (2006). Extreme selection in humans against homeotic transformations of cervical vertebrae. Evolution.

[ref-19] Garrut NV (1994). Dental ontogeny of the woolly rhinoceros *Coelodonta antiquitatis* (Blumenbach, 1799). Cranium.

[ref-20] Garrutt NV (1990). Anomalii zubnoj sistemy scerstistogo nosoroga *Coelodonta antiquitatis* (Blum., 1799). Fauna mlekopit. i ptiz pozdn. plejstoz. i goloz. SSSR. Leningrad, Akademii Nauk SSSR.

[ref-21] Hillman-Smith KK, Owen-Smith N, Anderson JL, Hall-Martin AJ, Selaladi JP (1986). Age estimation of the white rhinoceros (*Ceratotherium simum*). Journal of Zoology.

[ref-22] Jeannotte L, Lemieux M, Charron J, Poirier F, Robertson E (1993). Specification of axial identity in the mouse: role of the Hoxa-5 (Hox1. 3) gene. Genes & Development.

[ref-23] Jorgensen KD (1998). Minipig in reproduction toxicology. Scandinavian Journal of Laboratory Animal Science.

[ref-24] Kahlke RD (1999). The history of the origin, evolution and dispersal of the late pleistocene Mammuthus-Coelodonta faunal complex in Eurasia (Large Mammals).

[ref-25] Leboucq H (1898). Recherches sur les variations anatomiques de la première côte chez l’homme. Archives de Biologie.

[ref-26] Leshchinskiy SV (2012). Paleoecological investigation of mammoth remains from the Kraków Spadzista Street (B) site. Quaternary International.

[ref-27] Leshchinskiy SV (2015). Enzootic diseases and extinction of mammoths as a reflection of deep geochemical changes in ecosystems of Northern Eurasia. Archaeological and Anthropological Sciences.

[ref-28] Li ZL, Shiota K (2000). Stage-specific homeotic vertebral transformations in mouse fetuses induced by maternal hyperthermia during somitogenesis. Developmental Dynamics.

[ref-29] Markova AK, Puzachenko AYu, Van Kolfschoten T, Van der Plicht J, Ponomarev DV (2013). New data on changes in the European distribution of the mammoth and the woolly rhinoceros during the second half of the Late Pleistocene and the early Holocene. Quaternary International.

[ref-30] McDonald JH (2014). Handbook of biological statistics.

[ref-31] Merks JHM, Smets AM, Van Rijn RR, Kobes J, Caron HN (2005). Prevalence of RIB anomalies in normal Caucasian children and childhood cancer patients. European Journal of Medical Genetics.

[ref-32] Middleton K, Fish DE (2009). Lumbar spondylosis: clinical presentation and treatment approaches. Current Reviews in Musculoskeletal Medicine.

[ref-33] Miller W, Drautz DI, Ratan A, Pusey B, Qi J, Lesk AM, Tomsho LP, Packard MD, Zhao F, Sher A, Tikhonov A, Raney B, Patterson N, Lindblad-Toh K, Lander ES, Knight JR, Irzyk GP, Fredrikson KM, Harkins TT, Sheridan S, Pringle T, Schuster SC (2008). Sequencing the nuclear genome of the extinct woolly mammoth. Nature.

[ref-34] Mol D, Post K, Reumer JWF, Van der Plicht H, De Vos J (2006). The Eurogeul—first report of the palaeontological, palynological and archaeological investigations of this part of the North Sea, the Netherlands. Quaternary International.

[ref-35] Moodley Y, Russa IRM, Dalton DL, Kotzé A, Muya S, Huabensak P, Bálint B, Munimanda GK, Deimel C, Setzer A, Dicks K, Herzig-Straschil B, Kalthoff DC, Siegismund HR, Robovský J, O’Donoghue P, Bruford MW (2017). Extinctions, genetic erosion and conservation options for the black rhinoceros (*Diceros bicornis*). Scientific Reports.

[ref-36] Narita Y, Kuratani S (2005). Evolution of the vertebral formulae in mammals: a perspective on developmental constraints. Journal of Experimental Zoology Part B.

[ref-37] Nyström V, Humphrey J, Skoglund P, McKeown NJ, Vartanyan S, Shaw PW, Lidén K, Jakobsson M, Barnes I, Angerbjörn A, Lister A, Dalén L (2012). Microsatellite genotyping reveals end-Pleistocene decline in mammoth autosomal genetic variation. Molecular Ecology.

[ref-38] Palma A, Carini F (1990). Variazioni dellàpofisi trasversa della settima vertebra cervicale: studio anatomo-radiologico su una popolazione “segregate”. Archivio Italiano di Anatomia e di Embrioligia.

[ref-39] Post K (2005). A Weischelian marine mammal assemblage from the southern North Sea. Deinsea.

[ref-40] Protero D (2005). The evolution of North American Rhinoceroses.

[ref-41] Räikkönen J, Vucetich JA, Vucetich LM, Peterson RO, Nelson MP (2013). What the inbred scandinavian wolf population tells us about the nature of conservation. PLOS ONE.

[ref-42] Reumer JWF, Ten Broek CMA, Galis F (2014). Extraordinary incidence of cervical ribs indicates vulnerable condition in Late Pleistocene mammoths. PeerJ.

[ref-43] Sawin PB (1937). Preliminary studies of hereditary variation in the axial skeleton of the rabbit. The Anatomical Record.

[ref-44] Schultz AH (1961). Vertebral column and thorax.

[ref-45] Schumacher R, Mai A, Gutjahr P (1992). Association of rib anomalies and malignancy in childhood. European Journal of Pediatry.

[ref-46] Schut PC, Cohen-Overbeek TE, Galis F, Ten Broek CM, Steegers EA, Eggink AJ (2016). Adverse fetal and neonatal outcome and an abnormal vertebral pattern: A systematic review. Obstetrical & Gynecologial Survey.

[ref-47] Starck D (1979). Vergleichende Anatomie der Wirbeltiere.

[ref-48] Steigenga MJ, Ruinard S, De Koning J, Helmerhorst FM, Tijssen AMI, Galis F (2006). Evolutionary conserved structures as indicators of medical risks: increased incidence of cervical ribs after ovarian hyperstimulation in mice. Animal Biology.

[ref-49] Stephanopoulos G, Garefalaki M-E, Lyroudia K (2005). Genes and related proteins involved in amelogenesis imperfecta. Journal of Dental Research.

[ref-50] Stuart AJ, Lister AM (2012). Extinction chronology of the woolly rhinoceros Coelodonta antiquitatis in the context of late Quaternary megafaunal extinctions in northern Eurasia. Quaternary Science Reviews.

[ref-51] Subasioglu A, Savas S, Kucukyilmaz E, Kesim S, Yagci A, Dundar M (2015). Genetic background of supernumerary teeth. European Journal of Dentistry.

[ref-52] Ten Broek CMA, Bakker AJ, Varela-Lasheras I, Bugiani M, Van Dongen S, Galis F (2012). Evo-devo of the human vertebral column: on homeotic transformations, pathologies and prenatal selection. Evolutionary Biology.

[ref-53] Van Strien NJ, Manullang B, Sectionov IW, Khan MKM, Sumardja E, Ellis S, Han KH, Boeadi PJ, Bradley Martin E (2008). Dicerorhinus sumatrensis. http://dx.doi.org/10.2305/IUCN.UK.2008.RLTS.T6553A12787457.en.

[ref-54] Varela-Lasheras I, Bakker AJ, Van der Mije S, Van Alphen J, Galis F (2011). Breaking evolutionary and pleiotropic constraints in mammals: on sloths, manatees and homeotic mutations. EvoDevo.

[ref-55] Wéry N, Narotsky MG, Pacico N, Kavlock RJ, Picard JJ, Gofflot F (2003). Defects in cervical vertebrae in boric acid-exposed rat embryos are associated with anterior shifts of hox gene expression domains. Birth Defects Research Part A.

[ref-56] Willerslev E, Davison J, Moora M, Zobel M, Coissac E, Edwards ME, Lorenzen ED, Vestergard M, Gussarova G, Haile J, Craine J, Gielly L, Boessenkool S, Epp LS, Pearman PB, Cheddadi R, Murray D, Brathen KA, Yoccoz N, Binney H, Cruaud C, Wincker P, Goslar T, Alsos IG, Bellemain E, Brysting AK, Elven R, Sønstebø JH, Murton J, Sher A, Rasmussen M, Rønn R, Mourier T, Cooper A, Austin J, Möller P, Froese D, Zazula G, Pompanon F, Rioux D, Niderkorn V, Tikhonov A, Savvinov G, Roberts RG, MacPhee RDE, Gilbert MT, Kjær KH, Orlando L, Brochmann C, Taberlet P (2014). Fifty thousand years of Arctic vegetation and megafaunal diet. Nature.

